# Editorial Notes

**Published:** 1908-09

**Authors:** 


### Editorial Notes.

**Gastric Surgery.** THE two papers on "Surgical aid in chronic ulcer of the stomach," the one by Dr. J. A. Nixon (page 217) and the other by Mr. C. A. Morton (page 207), recall the fact that gastric surgery is of only recent date, and that it was in this *Journal* that Mr. Nelson C. Dobson was one of the first surgeons to suggest the desirability of surgical interference in perforating ulcer of the stomach. A reference to his paper (vol. i. p. 198) will show that its author was in advance of his time when he expressed "his hope and belief that before long this disorder, which is so commonly fatal, will be robbed of some of its perils by the timely and well-directed efforts of the surgeon." The results which we now publish are such as must greatly exceed the expectations of the most sanguine in 1883.

**Royal Army Medical Corps.** IT is very gratifying to those who have done the work to find that their efforts are being recognised and gratefully acknowledged. With reference to the patriotism of the medical profession, the Director-General of the Army Medical Service, in his Report dated July 18th, makes the following statement :—

"I cannot attempt here to describe the tasks which have been placed on the shoulders of the members of the medical profession. All the efforts connected with the organisation of a medical service of the Territorial Force have been made by men who are leading busy lives while undertaking these additional and heavy burdens. It is on principals and professors of universities, lecturers in medical schools, physicians and surgeons of great civil hospitals, and more especially on the general practitioners throughout the country, that the whole burden of training to equip themselves for the work now falls, and for this purpose they are devoting both time and money. I feel it to be my duty, therefore, to draw your special attention to the aid which I have received from these most patriotic men, the whole body of the medical profession."

Locally we know that this comment has been well deserved, and that the following quotation from the Bristol *Times and Mirror* (August 5th), with reference to our own Division, gives such well-deserved commendation :—

"The new Bristol Royal Army Medical Corps, under the command of Lieut.-Colonel Prichard, are bearing themselves right worthily. They are a credit to the Territorial Army."

**British Medical Association.**

Though no great discovery or startling announcement marked the Sheffield meeting of the British Medical Association, much valuable work was brought forward. As to the representative meeting, the new constitution is working well. The wearisome discussions on the Charter are apparently at an end, for the meeting finally decided by a practically unanimous vote that immediate application should be made for it. A determined effort had been quietly made during the winter on the initiative of the late President, Dr. Davy, to bring into harmony the divergent opinions of last year, and after a detailed re-examination of the proposed Charter very few alterations were made.

Nothing was more remarkable than the thorough and temperate way in which great practical questions were thrashed out, such, for instance, as the Principles of Hospital Management and the Ethics of Consultation. A hearing was given to every objector, but the meeting quickly made up its mind as to what was just and practical, or what was unripe for decision at the present time.

One new departure was agreed to in the establishment of a department at the annual meetings for the discussion of Sociology, at which lay persons might speak as well as medical men. An instance of the value of the participation of laymen in certain debates was seen when the Lord Mayor and Sir John Bingham spoke on the smoke abatement question, and explained the position of the manufacturer.

The meeting will long be remembered for the proverbial Yorkshire hospitality which was shown to the visitors. The beautiful excursions in the neighbourhood, the great manufactories on view in the city, and the entertainments provided by the inhabitants, will not easily be forgotten. A large number of the representatives were invited to Buxton from Saturday to Monday, and well entertained at the chief hotels. There, too, the weather was brilliant. The splendidly-equipped baths and the hospital were inspected, and the waters tasted. Quick trains brought back the fortunate members to Sheffield after a restful experience of the delights of Buxton. The cause of the therapeutic activity of the springs is by no means clear. They contain, principally, carbonate of lime and common salt, with some free nitrogen and $CO_2$. The temperature is only 80°, yet the waters have long been and are resorted to by crowds of sufferers, with apparently great real benefit, while our own Hotwells, with water of a somewhat similar type, are entirely disused.

There was a large attendance of eminent foreigners, and the display of various academic robes, both when honorary degrees were conferred and upon other occasions, was remarkable. Perhaps the greatest enthusiasm was that called forth by the appearance of Colonel Bruce, the discoverer of the cause of Malta Fever, who received his honours amid general applause, and whose address was followed with close attention.

In the Public Health sections several local matters of great interest cropped up. A scheme for supplying dried milk to infants during the diarrhœa season gave the curious result that though it did not altogether always prevent epidemic diarrhœa, it produced a remarkable gain in weight, and wiped out chronic dyspepsia among the infants. Sheffield, too, was specially proud of the results of her smoke abatement, and claims more sunshine than any manufacturing town in England. We had, indeed, almost too much during our week. The boiler chimneys ten years ago emitted dark smoke for 15 to 50 minutes per hour; now they are cut down to about 2 minutes for each boiler, without loss to the manufacturer. This depends largely on improved stoking. There are, indeed, some processes where it is not yet possible to abolish smoke in spite of the efforts which are being made. The remarkable fact remains that very little is done in private houses to abate smoke or prevent the fumes of gas fires circulating in the house, while such great results have been obtained in manufactories. In Liverpool and St. Helens I saw the crusade against smoke going on. Even the great liners on the Mersey are compelled to reduce their smoke, and our steamer promptly stopped its black cloud when it reached a certain point in the river. Edmund Owen, in his lecture on dust and disease, also referred to the question of smoke as an irritant to the lungs, increasing their susceptibility to disease.

The question of the notification of births led to considerable controversy in the section between medical men. One cannot help feeling that the difficulty might have been avoided if the Act had simply ordered registration to take place in the first week after birth, and that a copy of the register should be given to the medical officer of health at once.

The beautiful buildings of the University afforded an excellent site for the lectures and discussions. Unfortunately, the acoustic properties of the Firth Hall did not seem to be satisfactory. The speeches and the lecture of the president were inaudible to a large number, and the hall devoted to the representative meeting was most trying for the same reason. Lanterns and the kinematograph were utilised in the sections. The pictures of Dr. Wilfrid Harris illustrating the tremors and gait in nervous diseases were excellent, and it is to be wished that a similar set could be found in every medical school for the instruction of students. Another lantern demonstration which excited great interest was that by Professor Neisser, the discoverer of the gonococcus, who has devoted his private fortune to research, and has recently returned from a three years' investigation of syphilis in Java, where, with the aid of the German Government, inoculation tests were carried out on the larger apes. Neisser is convinced that the spirochæta pallida is the source of the disease, though a complete demonstration is still unattained.

In the section on Medicine a very interesting paper was that of J. H. Pratt, of Baltimore, describing a method of home treatment of tuberculosis. The patients were formed into a society or class, which met periodically. Both in class and at home they were taught how to manage themselves. Tents were put up for them in their gardens or on their roofs, and the advantage of this steady drill and care was shown in the very large percentage of cures effected. Nathan Raw also gave an account of his work with bovine tuberculin. He claims to protect children from infection by the use of a serum obtained from tuberculous cattle. For the cure of pulmonary tuberculosis he employs bovine tuberculin, reserving Koch's tuberculin for surgical cases only.

As regards the Section of Surgery, the three days were all so full that it is difficult to summarise their proceedings. On Wednesday Sir Watson Cheyne and Mr. Harold Styles introduced a discussion on the diagnosis and treatment of cancer of the breast and were followed by many speakers. There was but little difference of opinion expressed on important facts, but the advisability of removing supra-clavicular glands, the necessity for the sacrifice of the pectoralis minor, and the ultimate prognosis after operation were the points which gave rise to the most interesting discussion. Professor Murphy, of Chicago, was very emphatic as to the absolute unjustifiability of employing chloroform as an anæsthetic.

Thursday was devoted to a most instructive series of papers on the indications for nephrotomy and nephrectomy, introduced by Dr. Newman of Glasgow. The possibility of saving suppurating kidneys by drainage, the diagnosis of early tubercle of the kidney, and, above all, the importance of the cystoscope were the chief points of interest.

The third day was almost overcrowded by papers on diverse subjects, including one by Professor Lucas-Championiére on

"Mobilisation of Fractures," by Professor Tillmans on "Cerebral Puncture," by Professor Willens on "Tarsectomy for Talipes." The only regret one felt was that the time was too short for adequate questions and discussions.

The Pathological Museum contained a very remarkable collection of specimens illustrating the surgery of the kidney and extra-uterine gestation, besides many other subjects of medical interest.

---

